# Methane Combustion Using Pd Deposited on CeO_x_-MnO_x_/La-Al_2_O_3_ Pellistors

**DOI:** 10.3390/ma13214888

**Published:** 2020-10-30

**Authors:** Ovidiu G. Florea, Adelina Stănoiu, Marin Gheorghe, Cornel Cobianu, Florentina Neaţu, Mihaela M. Trandafir, Ştefan Neaţu, Mihaela Florea, Cristian E. Simion

**Affiliations:** 1 Laboratory of Atomic Structures and Defects in Advanced Materials, National Institute of Materials Physics, Atomistilor 405A, 077125 Magurele, Romania; ovidiu.florea@infim.ro (O.G.F.); adelina.stanoiu@infim.ro (A.S.); 2 NANOM-MEMS SRL, G. Cosbuc 9, 505400 Rasnov, Romania; maringhe@nanom-mems.com (M.G.); cornel.cobianu@nanom-mems.com (C.C.); 3 Laboratory of Nanoscale Condensed Matter, National Institute of Materials Physics, Atomistilor 405A, 077125 Magurele, Romania; florentina.neatu@infim.ro (F.N.); mihaela.trandafir@infim.ro (M.M.T.); stefan.neatu@infim.ro (Ş.N.); 4 Laboratory of Multifunctional Materials and Structures, National Institute of Materials Physics, Atomistilor 405A, 077125 Magurele, Romania; mihaela.florea@infim.ro

**Keywords:** CH_4_ catalytic combustion, Pd deposited CeO_x_-MnO_x_/La-Al_2_O_3_, selective catalytic reduction, pellistors

## Abstract

Pd deposited on CeO_x_-MnO_x_/La-Al_2_O_3_ has been prepared as a sensitive material for methane (CH_4_) detection. The effect of different amounts (1.25%, 2.5% and 5%) of Pd loading has been investigated. The as prepared materials were deposited on Pt microcoils using a drop-coating method, as a way of developing pellistors operated using a Wheatstone bridge configuration. By spanning the operating temperature range between 300 °C and 550 °C, we established the linearity region as well as the maximum sensitivity towards 4900 ppm of CH_4_. By making use of the sigmoid dependence of the output voltage signal from the Wheatstone bridge, the gas surface reaction and diffusion phenomena have been decoupled. The pellistor with 5% Pd deposited on CeO_x_-MnO_x_/La-Al_2_O_3_ exhibited the highest selective-sensitivity in the benefit of CH_4_ detection against threshold limits of carbon monoxide (CO), sulfur dioxide (SO_2_) and hydrogen sulfide (H_2_S). Accordingly, adjusting the percent of Pd makes the preparation strategies of pellistors good candidates towards CH_4_ detection.

## 1. Introduction

As the primary component of natural gas, methane (CH_4_) is an odorless, highly flammable gas with an overall global warming potential even higher than carbon dioxide (CO_2_). Its concentration in the surrounding atmosphere is continuously increasing, mainly due to the petrochemical and oil industries, pipe leakage, accidental releases during gas conduction, storage, or distribution and also due to the anaerobic decomposition of animal waste [1,2,3,4]. Moreover, natural gas is the second most employed source of energy owing to its abundance and straightforward burning processes [5]. As a consequence, solutions are required for widespread monitoring of methane emissions. Accordingly, a large diversity of CH_4_ sensors, involving different technologies, have been developed [6]. Among them, pellistors have advantages related to the low fabrication costs and simplicity in operation, being typically used in a Wheatstone bridge arrangement where the out-of-balance voltage from the bridge is used as a signal measurement [7,8]. From the phenomenological point of view, CH_4_ reduces the oxygen species from the catalytic material surface, leading to an exothermic reaction translated into the pellistor signal. The signal amplitude depends on the type of catalyst and on the concentration of the flammable gas [9]. However, highly working temperatures are required in order to gain a measurable signal by the Wheatstone bridge [10]. Since 1962, different catalytic materials for pellistors have been developed [11]. Noble metal oxide catalysts highlight a higher catalytic activity compared to their metallic counterparts [12,13]. Being cheaper than Pt and Rh, Pd is commonly used as catalytic converter. Moreover, PdO was found to be the most active metal oxide from the group of the platinic metal oxides group involved in combustion reactions. Accordingly, catalytic materials consisting of porous alumina-supported palladium have been intensively used for the catalytic combustion of methane at temperatures below 700 °C thanks to the Al_2_O_3_ capability to distribute the noble metal on a large surface area [14,15]. To increase the efficiency of catalytic combustion of methane at temperatures below 500 °C and still benefit from the large specific area of alumina, their functionalization with chemically active ceramics like zirconia (ZrO_2_) [16] or ceria-zirconia has also been developed [17]. Recently, functionalization of porous Al_2_O_3_ support with ceria (CeO_2_)-manganese (MnOx) has shown enhanced catalytic activity for CO oxidation at temperatures below 100 °C [18,19,20]. CeO_2_ can improve the performance of the sensor due to its high oxygen storage capability and redox properties [21]. Due to their ability to reversibly store oxygen and the propensity to stabilize transition metals in high dispersion render, CeOx represents a useful support for gas oxidation, while MnOx with its multiple oxidation states and weak oxygen bonds will contribute to the easy oxidation of CO [22]. On the other hand, ceria and manganese are widely used as oxidation catalysts also due to of the presence of oxygen vacancies from within [23]. As manganese possesses complex valences, it was thought to be a more active dopant then ceria for the redox property enhancement. The catalytic properties of MnOx are also known due to the oxygen storage capacity in their crystalline lattice [24]. Previous studies have shown the positive impact of CeOx-MnOx oxides on the oxidation reactions [25]. Only a few studies have reported the evaluation of the chemical activity of CeO_2_-MnOx in the catalytic combustion of methane at low temperatures [26]. For instance, excellent catalytic performances of the unsupported CeOx-MnOx binary composite in the CH_4_ oxidation have been shown with about 90% CH_4_ conversion (to CO_2_ and H_2_O) for temperatures higher than 390 °C for a modified co-precipitation method. The surface area of the catalyst was in the range of (70–100) m^2^/g, which was a good result, but below the surface area of the commercial porous Al_2_O_3_ which is in the range 150–180 m^2^/g. For the improvement of the surface area of the CeO_2_-MnOx binary catalyst, and the corresponding dispersion of the catalytic active centers on the surface, the chemical activity of the porous γ-Al_2_O_3_-supported CeO_2_-MnOx catalysts has been studied [27]. The catalytic combustion has shown a 90% CH_4_ conversion at temperatures higher than 450 °C. More recently, CH_4_ catalytic combustion on MnOx-CeO_2_ composites with different molar fractions of the CeO_2_ and MnOx has been investigated and showed 100% CH_4_ conversion at temperatures around 600 °C [28]. It is predictable that CeO_x_-MnO_x_/La-Al_2_O_3_ will have superior catalytic performance towards methane combustion, rather than a simple oxide mixture, most probably attributed to the increase of in active sites involved in catalytic reactions due to the large specific area of alumina. Moreover, CeOx in combination with Pd may result in low-temperature oxidation activity, Pd/CeOx being an effective catalyst for methane combustion, mainly from natural-gas powered combustion processes [29].

Knowing that CH_4_ has strong chemical bonds and needs high temperatures to break them, this study is focused on the catalytic performances of Pd-doped CeO_x_-MnO_x_/La-Al_2_O_3_ used as a pellistor’s sensitive element with selective sensitivity [30] in methane detection.

## 2. Materials and Methods

### 2.1. Pd Deposited on CeO_x_-MnO_x_/La-Al_2_O_3_ Pellistors

The sensitive material for methane combustion was synthesized in two steps: first, CeO_x_-MnO_x_/La-Al_2_O_3_ was prepared by hydrothermal method followed by the impregnation of this material with Pd, obtained by chemical reduction.

#### 2.1.1. CeO_x_-MnO_x_/La-Al_2_O_3_ Synthesis by Hydrothermal Method

All chemicals used in the preparation of catalytic materials are commercial and have been used without additional purification. The following chemical substances were used: cerium (III) nitrate hexahydrate (99.5% purity), manganese (II) nitrate tetrahydrate (both purchased from Acros Organics, Fair Lawn, NJ, USA), PdCl_2_ (99.9% purity) from Sigma-Aldrich (St. Louis, MO, USA). Commercially available Sasol Puralox TH100 (composition: Al_2_O_3_ with 4% La_2_O_3_) was used in this study as support for CeOx and MnOx.

Deionized water (18 MΩ × cm resistivity) was used in all the preparation steps. First, Puralox TH100 was dispersed in deionized water, followed by the dissolution of manganese and cerium nitrate salts. The molar ratio Ce/Mn/Al was 7/3/10. The pH was adjusted at 9 pH units with 25% aq. NH_4_OH. The mixture was heated in an autoclave 3 h at 180 °C, filtered, washed, and calcined 6 h at 500 °C (heating ramp of 6 °C/min.) After calcination, the material was grinded.

#### 2.1.2. Pd Deposition on CeO_x_-MnO_x_/La-Al_2_O_3_

Pd was deposited on the support by using wet impregnation. For this, PdCl_2_ was dissolved in HCl obtaining a clear solution and mixed with CeO_x_-MnO_x_/La-Al_2_O_3_, prepared previously. The quantities were dosed so that the Pd concentration of this mixture was 1.25%, 2.5% and 5%, respectively, by mass of the oxidic mixture. A reducing agent (hydroxylamine hydrochloride 99.995% purity, purchased from Sigma-Aldrich (St. Louis, MO, USA)) was added to every solution (double number of mol vs. PdCl_2_ quantity) in order to obtain Pd in metallic state. The water was removed by evaporation and finally calcined 1h at 500 °C, ground, and dispersed in terpineol (10% by mass percent).

#### 2.1.3. Pellistor Preparation

**Figure 1:**
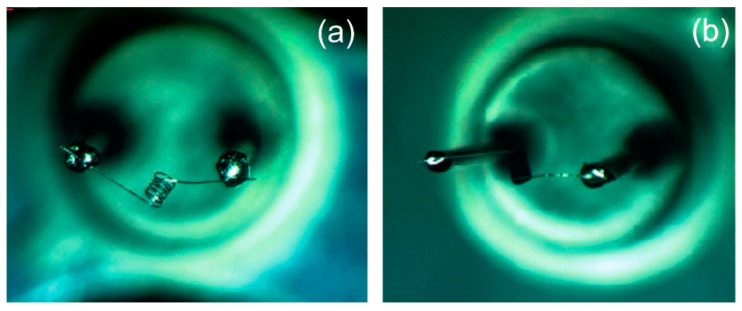
Pt microcoil (**a**) and the sensitive material deposited on microcoil ready for methane detection (**b**).

The as-prepared materials were deposited on Pt microcoils (made of platinum microwires of 25 µm diameter) by the drop method (see Figure 1a). The obtained pellistor is presented in Figure 1b. The samples were named as: D2 (CeO_x_-MnO_x_/La-Al_2_O_3_); 1,25%Pd/D2 (1.25%Pd deposited on CeO_x_-MnO_x_/La-Al_2_O_3_); 2.50%Pd/D2 (2.50%Pd deposited on CeO_x_-MnO_x_/La-Al_2_O_3_) and 5.00%Pd/D2 (5.00%Pd deposited on CeO_x_-MnO_x_/La-Al_2_O_3_).

### 2.2. Materials’ Characterization

To obtain information about the properties of the as-prepared samples, several characterization techniques were employed in this study. Thus, the morphological and vibrational properties, crystallinity of the samples, and type of crystalline phases present in the material were verified by scanning electron microscopy (SEM), Raman spectroscopy and powder X-ray diffraction (XRD). The Pd content was measured by inductively coupled plasma mass spectrometry (ICP-MS).

The X-ray diffraction measurements were realized by using a Bruker-AXS D8 Advance diffractometer (Bruker Corporation, Billerica, MA, USA.) equipped with a LynxEye 1D detector and Cu-Kα (0.1541 nm) radiation source and a scintillation counter detector. The diffraction patterns were recorded over a 2θ range of 10–80° with a 0.01° step size and using a counting time of 1 s per point. The identification of the XRD phases present in the samples was performed by using the Powder Diffraction File from the International Centre for Diffraction Data (PDF-ICDD). The surface morphology and the chemical composition were examined at room temperature by SEM and energy-dispersive X-ray spectroscopy (EDX) in a Zeiss Evo 50 XVP microscope (Oberkochen, Germany). The images were taken at 3000× (for the support) and 10,000× magnification (for the samples containing Pd) at a 20 kV acceleration voltage using the secondary electrons (SE) detector.

All Raman measurements were performed in the range between 100 and 700 cm^−1^ on a LabRAM HR evolution spectrometer (Horiba Jobin Yvon, Kyoto, Japan) equipped with an air-cooled charge coupled device (CCD) and a He-Ne laser (633 nm). All the Raman spectra were recorded at room temperature in the extended scan mode with an acquisition time of 10 × 30 s.

To determine the total amount of Pd, the solid samples were digested in a mixture of 40 vol.% aqueous HCl and HNO_3_ and then, for detection of the signal intensity of the palladium ions, the resulting solutions were introduced into the PlasmaQuant MS Elite ICP-MS system from Analytik Jena AG (Überlingen, Germany) equipped with a double-pass Scott-type spray chamber (Peltier cooled at 3 °C), a concentric glass nebulizer and Cetac ASX-560 (Cetac Technologies, Omaha, NE, USA) autosampler. Calibration solutions were prepared by dilution of a palladium certified reference material solution containing 1000 mg L^−1^ of Pd (certified value and uncertainty 1000.2 ± 3 mg L^−1^) purchased from CPA Chem (Stara Zagora, Bulgaria).

### 2.3. Working Principle of the Pellistor in a Wheatstone Bridge

**Figure 2:**
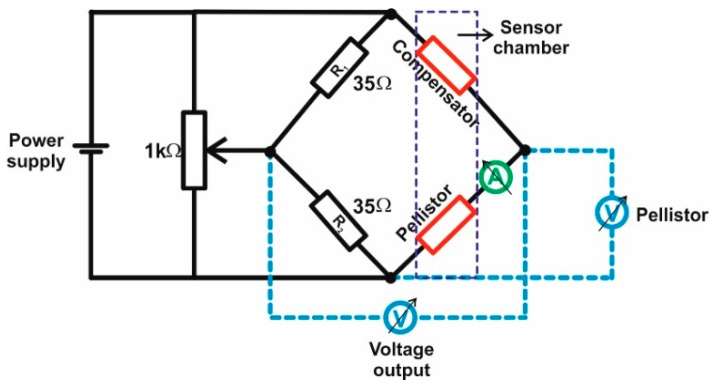
Wheatstone bridge setup used in evaluation of the catalytic performances of Pd deposited on CeO_x_-MnO_x_/La-Al_2_O_3_ pellistors.

The pellistor is usually made of thick and porous ceramic bead capable of detecting gases, which are combustible in air. It is designed to respond to a specific gas, strongly related to its specific properties (molecular weight, heat of oxidation, etc.). In our case, the CeO_x_-MnO_x_/La-Al_2_O_3_ bead was deposited on Pt microcoil in order to detect CH_4_. To reduce the activation energy of the combustion reaction, a metal catalyst (in our case Pd) is usually dispersed on the bead of the pellistor. For the catalytic performances evaluation, a power supply powers both arms of the Wheatstone bridge (see Figure 2). The balance voltage, measured to the output of the Wheatstone bridge, can afterwards be converted to CH_4_ concentration. The power supply heats the coils of the pellistor and compensator so that the both beads are raised to a temperature in the region of 500 °C. In the presence of combustible gas, the catalyst within the bead element enhances the gas to combust. The heat of combustion raises the temperature of the platinum coil, leading to an increase in the pellistor electrical resistance and the voltage drop across it. This unbalance the bridge and a useful output voltage can be measured.

## 3. Results

### 3.1. Materials’ Characterization

#### 3.1.1. X-ray Diffraction Analysis

**Figure 3:**
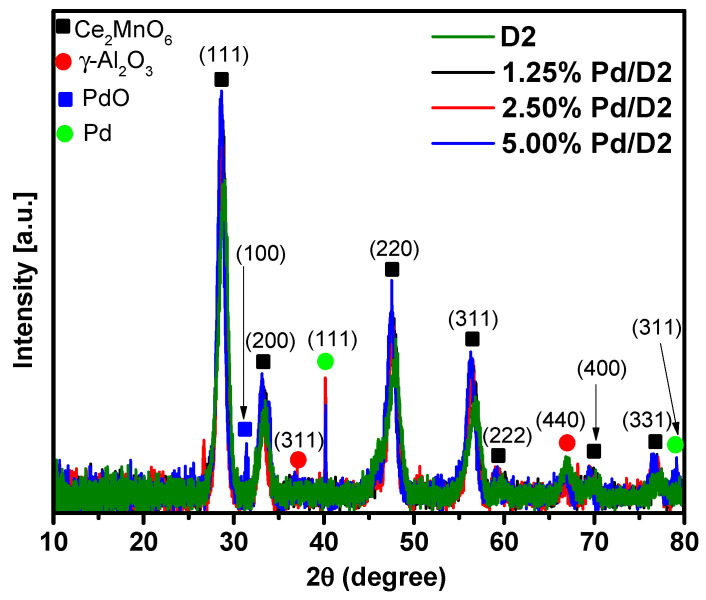
The X-ray diffraction (XRD) patterns of the as prepared Pd deposited on CeO_x_-MnO_x_/La-Al_2_O_3_ materials. The peaks of the Ce_2_MnO_6_ (00-064-0204), γ-Al_2_O_3_ (00-029-0063), tetrahedral PdO (00-041-1107) and Pd^0^ (00-001-1312) crystalline planes are labelled and indexed.

The XRD patterns of the all prepared materials are displayed in Figure 3. As the calcination temperature was just 500 °C, all materials are characterized by a low crystallinity. For the D2 sample, preponderantly Ce_2_MnO_6_ phase (PDF card 00-064-0204) with diffraction lines 2θ at about 28.6, 33.7, 47.5 and 56.9° can be observed, while only low intensity diffraction lines 2θ at about 59.6 and 66.9° can be associated with the existence of γ-Al_2_O_3_ cubic structure (PDF card 00-029-0063) from the Sasol-Puralox TH100 commercial material used in the preparation of this sample [31]. The absence of any detectable MnO_x_ phase suggests the formation of a Ce-O-Mn solid solution or that MnO_x_ is highly dispersed on the surface of all samples [32]. A closer look at the XRD patterns of the Pd deposited on D2 samples shows the appearance of new diffraction lines at 2θ of about 31.4, 40.1 and 79.4°, which can be assigned to the t-PdO(100) (PDF card 00-041-1107) [33,34] and also to the Pd^0^(111) and Pd^0^(311) crystalline planes (PDF card 00-001-1312), respectively [35,36]. This indicates that Pd deposition by impregnation method was successful. The presence of PdO, on the sample containing 5% Pd, can be beneficial for the methane combustion, as is presented further. Other diffraction lines typically assigned to the (200) and (222) crystalline planes of the face-centered cubic structure of palladium cannot be evidenced as they are overlapped by the strong peaks of both Ce_2_MnO_6_ and γ-Al_2_O_3_ predominant phases.

#### 3.1.2. Raman Spectroscopy

**Figure 4:**
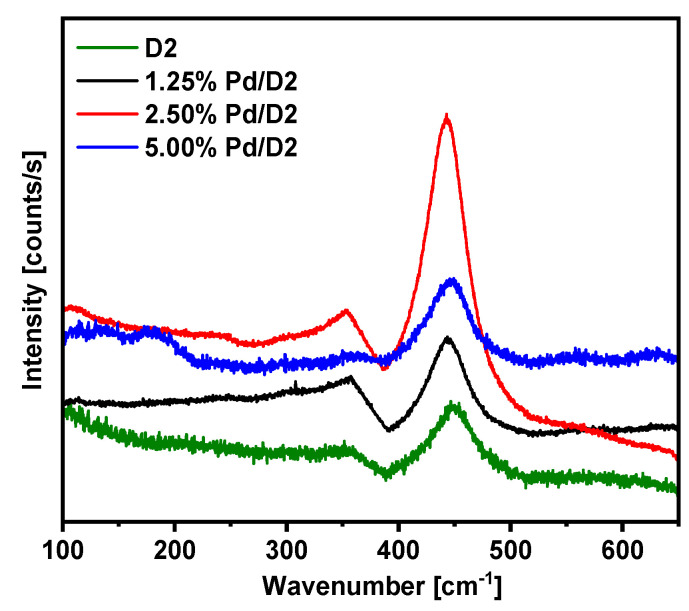
Raman spectra of the as prepared Pd deposited on CeO_x_-MnO_x_/La-Al_2_O_3_ materials.

The Raman spectra of all prepared materials are presented in Figure 4. As shown for the D2 sample, the strong Raman band centered at around 450 cm^−1^ might be associated to the F2g symmetric O-Ce-O vibration mode of the fluorite-type structure [37,38]. However, as the Raman characteristic band for pure CeO_2_ appeared at ca. 465 cm^−1^, such a big red shift of the F2g band can be attributed to the enlargement of the Ce-O bond lengths [39] as a result of lattice distortion with the formation of a Ce-O-Mn solid solution. Moreover, the absence of MnO_x_, already envisaged by XRD, and γ-Al_2_O_3_ bands is an indication of successful dispersion of Ce-O-Mn solid solution onto the surface of the alumina Sasol-Puralox TH100 commercial material support [40].

The deposition of Pd on the surface of D2 sample, independently of the loading, doesn’t modify the position of the 450 cm^−1^ Raman band and no other additional Raman peaks appear. This indicates that, as expected, the impregnation method does not affect the structural characteristics of the support.

#### 3.1.3. Scanning Electron Microscopy

**Figure 5:**
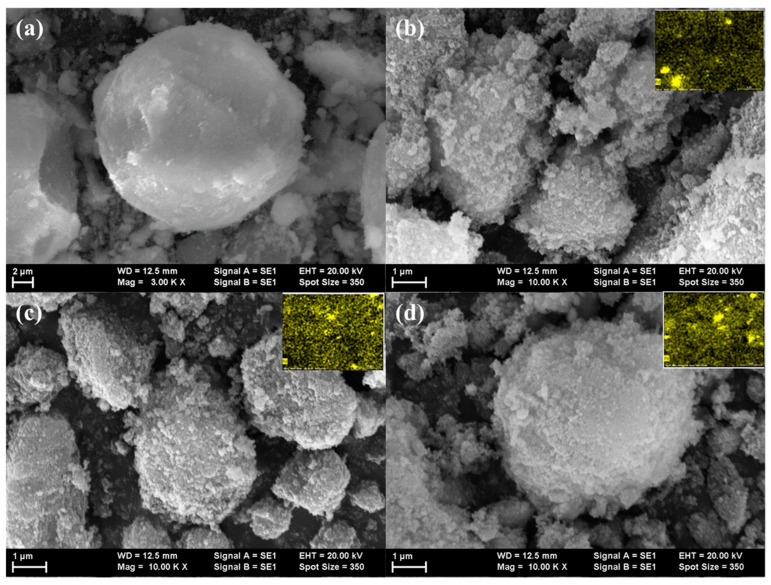
Scanning electron microscope (SEM) images for the investigated materials: (**a**) D2, (**b**) 1.25% Pd/D2, (**c**) 2.50% Pd/D2 and (**d**) 5.00% Pd/D2. In the up-right corner are depicted the mapping of Pd particles.

SEM images indicating the morphology of the investigated samples are presented in Figure 5. The D2 sample is preponderant, composed from very large spherical particles of about 10 μm, with a rather smooth texture. Further deposition of Pd changes the morphology. Smaller particles of irregular shapes of about 1–6 μm are formed, covered with spherical nanoparticles, with diameter smaller than 200 nm, independent of the Pd concentration.

The elemental composition of the D2, 1.25% Pd/D2, 2.50% Pd/D2 and 5.00% Pd/D2 (calculated from EDX spectra) is shown in Table 1. As can be observed, the concentration of the oxygen increased after Pd deposition on the support. This can be attributed to the formation of palladium oxide on the surface in agreement with X-ray diffraction data. The amount of Pd determined by EDX analysis is a little higher than the theoretical values, except the 2.5%Pd/D2 catalyst, for which the Pd amount is smaller (1.73%). Also, in Figure 5 are depicted the Pd particles distribution onto the surface of oxides. The elemental mapping evidenced that Pd forms small clusters on the support using the impregnation method.

The quantitative assessment of Pd was performed using ICP-MS analysis and the results are presented in Table 1. The values determined are close to the theoretical ones (±1.2%).

### 3.2. CH_4_ Catalytic Combustion Results

**Figure 6:**
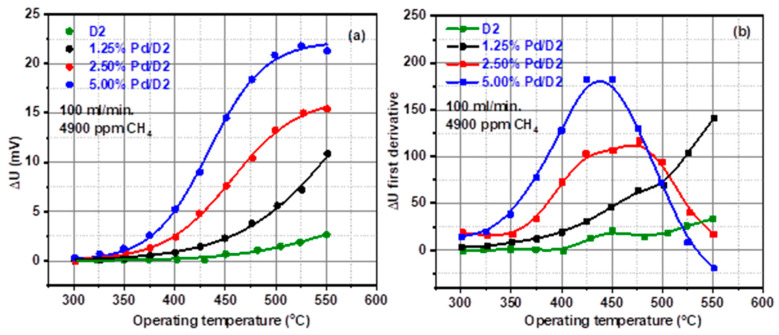
Voltage signal as a function of pellistor operating temperature at constant gas concentration (**a**) and the first derivative of voltage measured by the Wheatstone bridge (**b**).

The effect of the operating temperature on the output voltage of a typical pellistor element is shown in Figure 6a. As can be seen, all the curves were nicely fitted using sigmoid functions. With the increase in Pd concentration, the slope tangent to the curve is moving towards lower temperatures, meaning that the rate of combustion is dependent on the amount of Pd loading [41].

Moreover, at higher temperatures the response has a lower dependence on the operating temperature approaching a plateau region. This aspect is in good agreement with T.A. Jones and P.T. Walsh as a limited mass transport of fuel and to its optimal reaction balance between fuel and oxygen [42].

The temperature of the pellistor increases in the presence of CH_4_ and this results in the overall pellistor response. The higher the operating temperature is, the lower the influence of the surrounding atmospheric conditions is [43]. The behavior plotted in Figure 6b indicate that with the increase in the Pd amount, the peak level decreases with respect to the operating temperature.

As can be observed in the Figure 6b, the pellistor containing 5% Pd exhibits the lowest operating temperature (425 °C) for methane combustion. This is correlated well with the presence of a higher PdO amount on the surface of the 5% Pd/D2 pellistor, evidenced by X-ray diffraction patterns.

**Figure 7:**
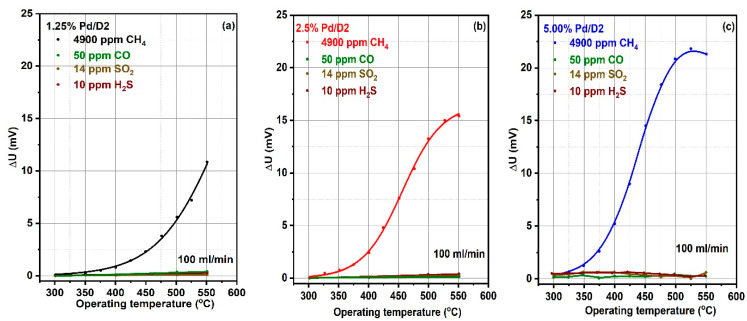
Selective-sensitivity plot of 1.25% (**a**), 2.5% (**b**) and 5% (**c**) Pd deposited on CeO_x_-MnO_x_/La-Al_2_O_3_ with respect to different target gases.

Selectivity and linear response are among the most important parameters of a sensitive material when it comes to application potential. In this respect, we have tested the pellistors loaded with 1.25%, 2.5% and 5% Pd. As can be seen in Figure 7a–c the tested pellistors exhibit the highest response in the benefit of CH_4_ detection against other potential interfering gases (CO, SO_2_ and H_2_S) when tested under the same working conditions. The gas levels have been selected according to the European Union (EU) imposed limits. 

### 3.3. Modeling of the CH_4_ Catalytic Combustion

From the experimental point of view, we established that the voltage signal from the Wheatstone bridge (as a function of detector operating temperature at constant gas concentration) was higher for the highest amount of Pd nanoparticle catalytic layer.

**Figure 8:**
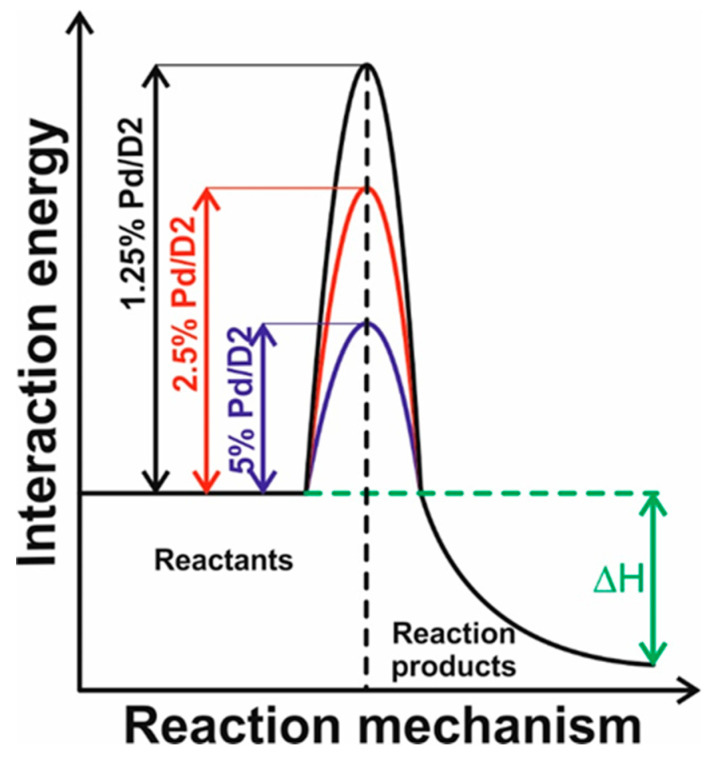
Schematic approach aiming to explain the role of %Pd towards improving the CH_4_ detection properties of CeO_x_-MnO_x_/La-Al_2_O_3_.

It is known that the Pd catalyst is required to reduce the activation energy, which is the minimum energy required in order for a reaction to proceed (see Figure 8). The activation energy, represents the potential energy barrier between reactants and products that must be overcome for the reaction to take place and is linked to the energy of a transition state that exists in the reaction mechanism. In our case the amount of Pd catalyst lowers the energy of the transition state and, therefore, lowers the activation energy, allowing the reaction to proceed at lower temperature. The chemical reaction between the reactants (CH_4_ and oxygen) is accomplished in contact with the catalyst surface forming the combustion products, while the Pd catalyst is not consumed.

Since the energy to drive the reaction consists in the heat generated by the platinum coil, the need of Pd catalyst allows the reaction to proceed at lower electrical power. As the voltage across a pellistor is increased, the energy of the catalyst increases to a sufficient energy level to become available to combust the CH_4_ over the pellistor, leading to a readable signal. With increasing voltage, this signal reaches a plateau region where the heat from the combustion is balanced by the heat exchanges with the surrounding atmosphere.

**Figure 9:**
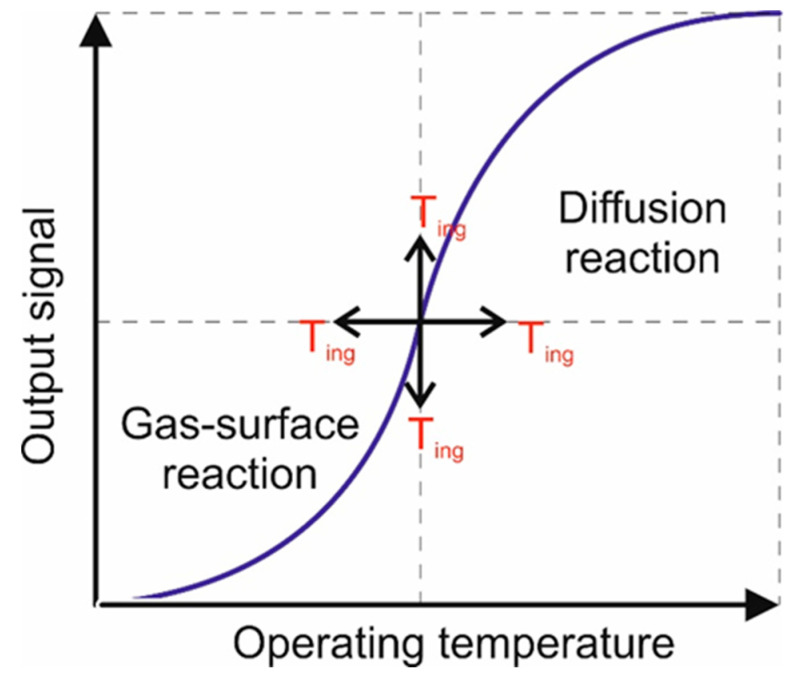
Schematic approach aiming to explain the sigmoid behavior of the 5% Pd deposited on CeO_x_-MnO_x_/La-Al_2_O_3_ with respect to the operating temperature.

An additional increase in voltage is translated into heat loss, reflected through a decrease in the sensor signal. As can be seen from the experimental results, a plot of this response for a fixed gas concentration versus operating temperature is commonly a sigmoid curve (Figure 9).

Such behavior can be described via two major step processes. At low operating temperature the main detection mechanism corresponds to a surface reaction control, whereas for the higher operating temperature regime diffusion transfer control is likely to occur. This performance was reported also by D. Shlenkevitch et al. [44].

The ignition temperature (denoted below by T_ing_) is defined as the transition temperature between these two regimes. The ignition temperature relates to the maximal change of released power as a function of temperature, and it is specific for the analyte–gas and catalytic–layer combination.

## 4. Conclusions

In this paper we have studied the role induced by the presence of Pd over the surface of CeO_x_-MnO_x_/La-Al_2_O_3_ towards CH_4_ detection performance. Accordingly, different amount of Pd (1.25%, 2.5% and 5%) was deposited over the surface of the “host” material in terms of CeO_x_-MnO_x_/La-Al_2_O_3_. The characterization techniques employed have demonstrated that the impregnation method is suitable for such materials, and the support preserves its initial characteristics.

The built pellistors were subject to CH_4_ detection over a wide range of operating temperature regimes. In this respect we could determine the linearity region of operation, as well as the maximum temperature for the highest value of the signal output from the Wheatstone bridge. The highest catalytic conversion of methane was attained using 5% Pd deposited on CeO_x_-MnO_x_/La-Al_2_O_3_, most probably in the presence of two types of Pd, cationic and metallic. Using the aforementioned material, we have emphasized two major characteristics, namely the linearity region with respect to different concentrations of CH_4_ as well as its higher selective potential manifested against different target gases such as, CO, SO_2_ and H_2_S.

In accordance with other literature, we have presented a schematic approach aiming to explain the sigmoid behavior expressed by the 5% Pd deposited on the CeO_x_-MnO_x_/La-Al_2_O_3_ pellistor with respect to the surface reaction and bulk diffusion.

## Figures and Tables

**Table 1:** Elemental composition of the investigated samples.

EDX	Normalized Mass (%)			
	D2	1.25% Pd/D2	2.50% Pd/D2	5.00% Pd/D2
C	13.80	13.61	13.01	11.10
O	33.92	53.12	49.63	46.17
Al	23.72	11.76	14.73	15.52
Mn	3.98	2.38	2.71	2.49
La	1.67	0.88	0.87	0.93
Ce	22.91	16.75	17.32	18.21
Pd	-	1.50 (1.38) ^1^	1.73 (2.21) ^1^	5.58 (5.61) ^1^
Total	100	100	100	100

^1^ The numbers from the brackets correspond to the palladium concentration determined by inductively coupled plasma mass spectrometry (ICP-MS) analysis.
